# Peripheral neuropathy, an independent risk factor for falls in the elderly, impairs stepping as a postural control mechanism: A case‐cohort study

**DOI:** 10.1111/jns.12656

**Published:** 2024-09-01

**Authors:** Felix Kohle, Christopher Stark, Heinz‐Dieter Klünter, Daniel Wernicke, Gilbert Wunderlich, Gereon R. Fink, Jens P. Klussmann, Michael Schroeter, Helmar C. Lehmann

**Affiliations:** ^1^ Department of Neurology, Faculty of Medicine University of Cologne and University Hospital Cologne Cologne Germany; ^2^ Department of Otorhinolaryngology, Head and Neck Surgery, Faculty of Medicine University of Cologne and University Hospital Cologne Cologne Germany; ^3^ Cognitive Neuroscience, Research Center Juelich Institute of Neuroscience and Medicine (INM‐3) Juelich Germany; ^4^ Department of Neurology Hospital Leverkusen Leverkusen Germany

**Keywords:** aging, frailty, geriatrics, posturography, proprioception

## Abstract

**Background/Aims:**

Peripheral neuropathies perturbate the sensorimotor system, causing difficulties in walking‐related motor tasks and, eventually, falls. Falls result in functional dependency and reliance on healthcare, especially in older persons. We investigated if peripheral neuropathy is a genuine risk factor for falls in the elderly and if quantification of postural control via posturography is helpful in identifying subjects at risk of falls.

**Methods:**

Seventeen older persons with a clinical polyneuropathic syndrome of the lower limbs and converging electrophysiology were compared with 14 older persons without polyneuropathy. All participants were characterized via quantitative motor and sensory testing, neuropsychological assessment, and self‐questionnaires. Video‐nystagmography and caloric test excluded vestibulocochlear dysfunction. For further analysis, all subjects were stratified into fallers and non‐fallers. Overall, 28 patients underwent computerized dynamic posturography for individual fall risk assessment. Regression analyses were performed to identify risk factors and predictive posturography parameters.

**Results:**

Neuropathy is an independent risk factor for falls in the elderly, while no differences were observed for age, gender, weight, frailty, DemTect test, timed “Up & Go” test, and dizziness‐related handicap score. In computerized dynamic posturography, fallers stepped more often to regain postural control in challenging conditions, while the Rhythmic Weight Shift test showed a lack of anterior‐posterior bidirectional voluntary control.

**Interpretation:**

Our study confirms peripheral neuropathy as a risk factor for older persons' falls. Fallers frequently used stepping to regain postural control. The voluntary control of this coping movement was impaired. Further investigations into these parameters' value in predicting the risk of falls in the elderly are warranted.

**Abbreviations**
CI: confidence interval
CIDP: chronic inflammatory demyelinating polyneuropathy
CMAP: compound muscle action potential
DCL: bidirectional control
DHI: Dizziness Handicap Inventory
DML: distal motor latency
FES: Falls Efficacy Scale
ICD: International Statistical Classification of Diseases and Related Health Problem
mCTSIB: modified Clinical Test of Sensory Interaction in Balance
MRC: Medical Research Council
mRS: modified Rankin Scale
NCV: nerve conduction velocity
OR: odds ratio
SNAP: sensory nerve action potential
SOT: Sensory Organization Test

## 1 INTRODUCTION

Postural control involves a complex interplay of sensory and motor networks on different levels of the peripheral and central nervous systems to maintain the center of mass over its base of support, primarily in humans the lower extremities, enabling upright walking.^1^ Several sensory receptors convey information about the environment, for example, Meissner corpuscles detect light touch, and Ruffini corpuscles tune to skin stretch, allowing the perception of changes of the body's surface. By contrast, muscle and joint proprioceptors detect position and movement in space and are essential for agonist‐antagonist synchronization.^2^, ^3^ Postural adjustments are made based on integrated visual and vestibular information, often without conscious interference.^3^ The Romberg sign is an excellent clinical example: patients with postural instability start swaying when they close their eyes because the visual system can no longer subconsciously correct the instability.^4^ Notably, the postural control network adjusts to rapid and slow perturbations of the subject's environment and the system itself.^5^

However, why, in some older persons, the control is lost with subsequent risk for falls, whereas others can compensate for disease‐related dysfunction is not well understood. Falls in the elderly considerably impact quality of life and morbidity.^6^, ^7^ A detailed diagnostic assessment is indicated to recognize possible treatable causes at an early stage and initiate physiotherapy for fall prevention.^8^, ^9^ The disposition to falls is influenced by various factors and diseases, which often potentiate each other, making fall risk assessment in older persons challenging, constituting a critical problem in geriatric settings.^10^ The frailty syndrome has been defined to assess the compensatory capacity of geriatric patients to cope with disease‐related stressors and identify patients at risk for falling.^11^, ^12^ As described by Fried et al.,^12^ self‐reported fatigue, weakness, slow walking speed, and low physical activity are four (of five) criteria to identify frail patients. Notably, these criteria are also frequent symptoms of peripheral neuropathy. Further, these criteria have also been incorporated in the LUCAS Frailty Index, a patient‐reported score assessing risk factors and functional resources.^13^

To sum up, peripheral neuropathy, the most prevalent neurological disorder in older persons, impacts both frailty and falls.^14^ The postural control of neuropathy patients is impaired through decreased sensory acuity, including impaired touch sensation and proprioception, and muscle strength. Several retro‐ and prospective studies addressed this interaction and suggested that peripheral neuropathy is a risk factor for falls.^15^, ^16^, ^17^, ^18^ However, definitions of peripheral neuropathy in these studies ranged from ICD code‐derived data,^15^ monofilament‐based definitions,^18^ to neuropathy of the lower limbs with verification by nerve conduction studies (albeit in a heterogeneous group and with only limited reporting of other potential confounders).^17^ Furthermore, it remains to be elucidated why some patients with peripheral neuropathy can compensate for sensorimotor deficits while others are prone to falls, and whether it is possible to identify these patients at risk.^19^

Computerized dynamic posturography is a quantifiable tool for identifying patients at risk for falls and impaired gait stability. It is sensitive to several pathological conditions that influence gait stability, such as peripheral diabetic neuropathy and vestibular disorders.^20^, ^21^, ^22^, ^23^, ^24^, ^25^ However, its utility to predict the fall risk in older persons with peripheral neuropathy has not yet been shown.

Accordingly, we investigated a well‐defined cohort of home‐dwelling persons over 60 years of age with or without polyneuropathy of the lower limbs with a detailed clinical and demographic characterization (i.e., medical history, falls and their impact on daily activities and quality of life, memory, gait, and strength assessment). We assessed whether peripheral neuropathy is a genuine risk factor for falls and quantified the fall risk using computerized dynamic posturography to identify frequent fallers.

## 2 METHODS

**FIGURE 1:**
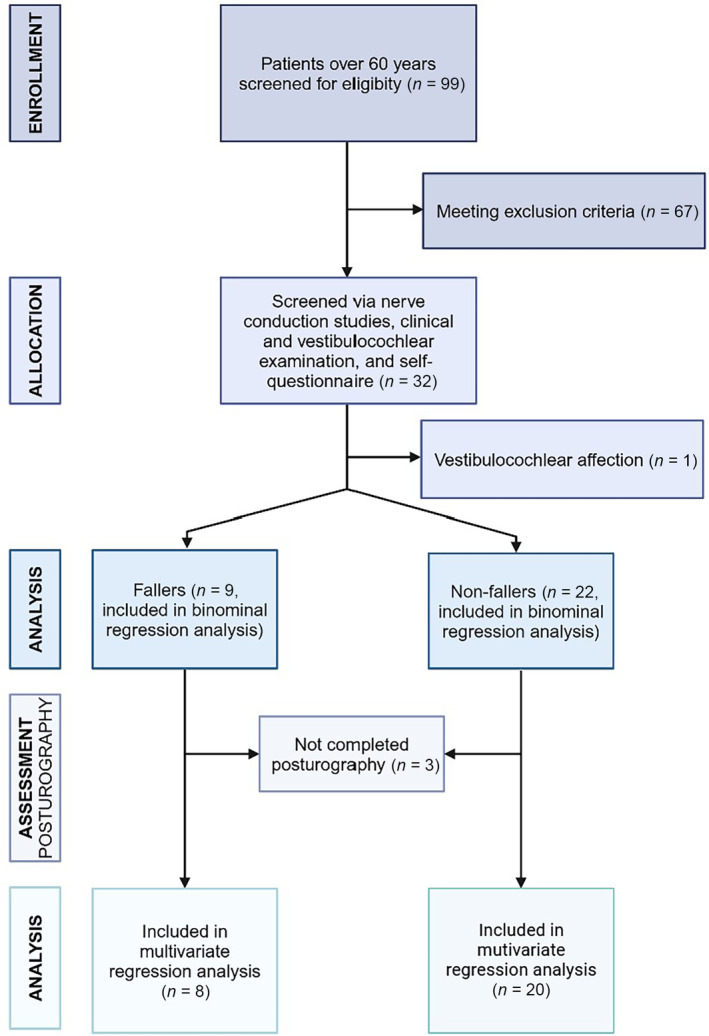
Flow chart depicting the study selection process. Created with BioRender.

All patients were recruited at the Department of Neurology at the University Hospital of Cologne between January 2022 and December 2023. We initially planned to include 18 patients in each group, based on a Cohens d‐effect of 0.5, an estimated self‐reported fall prevalence of 10% in the control and 40% in the peripheral neuropathy group, a drop‐out rate of 5%, and a two‐sided *t*‐test (alpha = .05, Power = 0.8), calculated by using the open‐source G‐Power Software.^26^ A cross‐sectional case‐cohort design (Figure 1) was used. The local ethic committee approved the study performed under the Declaration of Helsinki (21‐1447), and all patients gave written informed consent before participation.

### 2.1 Patient selection

Inclusion criteria were age over 60 years and an electrophysiological affection of at least two lower limb nerves for the polyneuropathy group and self‐reported sensory deficits of the lower limbs. Likewise, the control group showed no pathological nerve conduction studies and no sensory deficits of the lower limbs. Notably, hereditary neuropathies were excluded (Figure 1). Further, only patients with a modified Ranking Scale (mRS) <3^27^ and an unaided walking capability of >500 m were included. All participants were home‐dwelling older persons. Other exclusion criteria were: age under 60 years, other neurological degenerative diseases, orthopedical, or other disorders that might interfere with the study procedures (e.g., multiple sclerosis, Parkinson's disease, history of a stroke, surgery within the last month, lumbar herniated disc with subsequent paresis, spinal stenosis). Patients with a known affection of the vestibulocochlear system (e.g., neuritis vestibularis, benign paroxysmal positional vertigo) were also excluded. Further, we screened all patients for a subclinical affection of the vestibulocochlear system via video‐nystagmography, the head impulse test, and thermal (caloric) water irrigation, which led to the exclusion of one subject. All patients were subsequently grouped into fallers (≥1 self‐reported fall in the last year) and non‐fallers (no reported fall in the last year) to assess the risk for falls.

### 2.2 Neurological examination, including gait and sensorimotor assessment

All patients received a thorough neurological examination by a blinded, experienced examiner (FK or CS). Sensory acuity was assessed using a Rydel‐Seiffer tuning fork at the ulnar styloid process, interphalangeal joint of the hallux, and internal malleolus for the adapted vibration score.^28^ Position sensing in each patient was tested as described.^29^ The light touch sensation of each side's hallux was examined using two‐point discrimination. MRS,^27^ the modified Medical Research Council (MRC) sum score,^30^, ^31^ and the timed “Up & Go” test^32^ were performed. Standardized neurophysiological testing assessed the right tibial and sural nerves' compound muscle action potential (CMAP), sensory nerve action potential (SNAP), and nerve conduction velocity (NCV), respectively. The DemTect test was performed under standardized condition.^33^

### 2.3 Fall assessment via self‐questionnaires

Each patient filled out a nine‐page self‐questionnaire at the study inclusion, including the number of falls in the last year. We further asked for gait imbalance symptoms: recent stumbles, insecure gait, and false estimation of heights (e.g., stair steps). Current medication and comorbidities were retrieved from medical records. To compare comorbidities between the groups, we used the updated Charlson comorbidity index.^34^, ^35^ For assessing the functional capacity, the LUCAS Frailty Index was used.^13^ Whether falls (or the fear of falling) would impact daily‐life activities was investigated via the German version of the Falls Efficacy Scale (FES‐I).^36^ For a dizziness‐related impact, the Dizziness Handcap Inventory (DHI) was used.^37^

### 2.4 Dynamic computerized posturography

We assessed balance function and fall risk via the Balance Master System test battery (NeuroCom).^38^, ^39^ Blinded and experienced examiners (DW and H‐DK) performed the following tests:

1. the modified Clinical Test of Sensory Interaction on Balance (mCTSIB)^40^, ^41^ under four different testing situations: (a) eyes open, firm surface (Eo firm), (b) eyes closed, firm surface (Ec firm), (c) eyes open, unstable surface (Eo foam), and (d) eyes closed, unstable surface (Ec foam). For all testing conditions, the center of gravity sway trajectory in degree/s was determined based on force plate measurements, and a composite score was calculated.^42^ Steps to maintain balance were registered. Further, the Limit of Stability was calculated.^43^ It represents the maximum distance a participant can intentionally sway in any direction without losing balance or needing to take a step. It is also based on the center of gravity. A score of 100 represents no loss of balance, while lower scores imply a higher postural instability.^44^
2. The Sensory Organization Test (SOT) to assess how visual, vestibular, or somatosensory information to maintain an upright stance were used under six different sensory conditions: (SOT 1) open eyes with visual surroundings and the platform are stable; (SOT 2) closed eyes and stable platform; (SOT 3) open eyes with the surroundings moving in response to the body sway, (SOT 4) sway‐referenced platform, open eyes and stable surroundings, (SOT 5) closed eyes and sway‐referenced platform; and (SOT 6) sway‐reference of both the platform and surroundings. The SOT highest possible score of 100 indicates no sway at all, while the lowest score of 0 indicates a step to maintain balance. A SOT composite score was calculated by (a) independently averaging the equilibrium scores for conditions 1 and 2; and (b) adding these two scores to the equilibrium scores from each trial of sensory conditions 3, 4, 5, and 6 and dividing that sum by the total number of trials as described in References 40, 45.
  Further, the sensory analysis ratios were calculated as described in Reference 46:
  - somatosensory (SOM) for somatosensory input
  - visual (VIS) for visual input
  - vestibular (VEST) for vestibular input
  - preference (PREF) reliance on (incorrect) visual input.
3. Motor control tests. We applied two different motor control tests to evaluate compensation mechanisms in the patients: (a) the Rhythmic Weight Shift test, which assesses the voluntary ability of the participants to move the center of gravity from right to left and forward to backward by following a visualized dot between two visualized targets at three different velocities (slow, medium, and fast). The outcome variables were the on‐axis speed of the center of gravity movement and bidirectional control in percentage (ratio of the movement in the intended direction to the deviation from this ideal trajectory), calculated for every movement direction and speed.^43^ (b) The adaptation test for testing the motor control in response to sudden changes of the foot plate.^40^, ^47^

### 2.5 Vestibulocochlear function

As we wanted to define the role of peripheral neuropathy as a genuine risk factor for falls, we screened every patient for vestibulocochlear dysfunction with video‐nystagmography for spontaneous nystagmus and after positioning maneuver as well as caloric testing. The examiner was blinded (DW).

### 2.6 Statistics

Statistical analyses were performed using SPSS 29.0.2.0 for Windows (IBM, Armonk, NY, USA). We used chi‐square tests or Fisher's exact tests (if less than five items) to compare categorical independent data. Data were tested for Gaussian distribution using the Shapiro‐Wilk test. For non‐normally distributed data, we used the Mann‐Whitney test and reported median and interquartile range. For normally distributed data, *t*‐tests for unpaired variables were employed to compare independent metrical data and mean and standard deviation was reported. All tests were performed two‐tailed. Statistically significant differences in patient and therapy characteristics were further analyzed with logistic regression analysis for the primary outcome (falls, dependent variable). *p*‐Values <.05 were estimated as significant. A binominal logistic regression analysis for the dependent variable falls (0 = no, 1 = yes) to identify potential risk factors was then performed. To predict the individual fall risk, the number of overall falls as a dependent variable was used for a multivariable linear regression analysis, and postural control parameters generated by computerized dynamic posturography were used as independent variables. Results of the regression analyses were plotted using GraphPad Prism 10 (San Diego, CA, USA).

## 3 RESULTS

### 3.1 Selection process

We screened 99 patients for eligibility. Sixty‐eight patients met one or more exclusion criteria. Overall, 17 older patients with polyneuropathy of the lower limbs and 14 older controls were included and stratified for fallers (*n* = 9) and non‐fallers (*n* = 22). All patients underwent dynamic computerized posturography for postural control. Three patients did not complete the protocol and were excluded from further analysis. Reported drop‐out reasons were: lower back pain (*n* = 1), scheduling difficulties with another appointment (*n* = 1), while one patient gave no reason.

### 3.2 Patient characteristics

Detailed demographic data are provided in Table 1. For the demographic data of the neuropathy and control patients, see Supplemental material 1a–d. No significant differences in gender, age, or weight were seen between fallers and non‐fallers. However, nearly all fallers were neuropathy patients (89%), while only 41% of the non‐fallers had polyneuropathy (*p*‐value: .021). Fallers showed a higher mRS (median 1, *p*‐value: .022).

**TABLE 1:** Patient characteristics.

	Fallers (*n* = 9)	Non‐fallers (*n* = 22)	Significance
Sex (*n* of females)	3 (33%)	12 (55%)	0.43^a^
Age (mean in years, SD)	73.3, ±10.7	70.2, ±7.4	0.45^b^
BMI (mean, SD)	26.2, ±4.3	27.4, ±6.1	0.53^b^
Polyneuropathy	**8 (89%)**	**9 (41%)**	**0.021** ^a^
mRS (median, IQR)	**1, [1–2]**	**0, [0–1]**	**0.022** ^b^
Comorbidities (*n*)			
Congestive heart failure	0 (0%)	3 (33%)	
Hemiplegia or paraplegia	0 (0%)	0 (0%)	
Dementia	0 (0%)	0 (0%)	
Chronic pulmonary disease	0 (0%)	4 (18%)	
Rheumatological disease	3 (33%)	1 (5%)	
Diabetes with chronic complications	3 (33%)	2 (9%)	
Renal disease	0 (0%)	2 (9%)	
Any malignancy	1 (11%)	0 (0%)	
Metastatic solid tumor	0 (0%)	1 (5%)	
Liver disease, any	0 (0%)	0 (0%)	
Acquired immunodeficiency syndrome	0 (0%)	0 (0%)	
Charlson comorbidity index (max. 24, median, IQR)	0, [0–2]	0, [0–1]	0.78^b^
Medication (*n*)			
Antihypertensives	2 (22%)	13 (59%)	
Antipsychotics	0 (0%)	0 (0%)	
Antidepressants	1 (11%)	3 (14%)	
Anxiolytics	0 (0%)	0 (0%)	
Narcotics	0 (0%)	2 (9%)	
Nonsteroidal anti‐inflammatory drugs	0 (0%)	1 (5%)	
Antihistamines	0 (0%)	0 (0%)	
Anticholinergic drugs	0 (0%)	1 (5%)	
Antiepileptics	0 (0%)	3 (14%)	
Proton pump inhibitors	2 (22%)	3 (14%)	
N. suralis			
sNCV (median in m/s, IQR)	**0, [0–36.5]**	**43, [38.8–48.5]**	**0.005** ^b^
sSNAP (median in μV, IQR)	**0, [0–3.5]**	**6.5, [3.8–12.8]**	**0.003** ^b^
N. tibialis			
mNCV (median in m/s, IQR)	**38 [34–41.5]**	**45, [40–49]**	**0.015** ^b^
CMAP (median in mV, IQR)	**3, [0.5–5.2]**	**10.3, [6–17.2]**	**0.003** ^b^
DML (median in m/s, IQR)	**5.1, [4.6–6.9]**	**4, [3.5–4.7]**	**0.002** ^b^
Polyneuropathy subtype by NCS (*n*)			
Mainly axonal sensorimotor	3	14	
Mainly axonal sensory	0	4	
Axonal‐demyelinating sensorimotor	5 (56%)	4	

*Note*: *p*‐Values < .05 were estimated as significant and are highlighted in bold.

Abbreviations: BMI, body mass index; CMAP, compound muscle action potential; DML, distal motor latency; IQR, interquartile range; mNCV, motor nerve conduction velocity; mRS, modified Rankin Scale; NCS, nerve conduction studies; SD, standard deviation; SNAP, sensory nerve action potential; sNCV, sensory nerve conduction velocity.

^a^ Chi‐square test.

^b^ *t*‐Test/Mann‐Whitney test.

### 3.3 Neurological examination, including gait and sensorimotor assessment

Concordant to the high prevalence of neuropathy in the fallers group, a decreased sensory acuity was observed. The Romberg test was positive in 67% and only in 18% of the non‐fallers (*p*‐value: .015). Absent or weak reflexes were noted in 78% (compared with 27% of the non‐fallers, *p*‐value: .017). Furthermore, the vibration score, position sensing, two‐point discrimination, and MRC sum score were significantly reduced in the fallers group (see Table 2 for details). Notably, the timed “Up & Go” test—a reported sensitive clinical test to detect fallers—did not differ significantly between the groups.

**TABLE 2:** Neurological examination, including gait and sensorimotor assessment.

	Fallers (*n* = 9)	Non‐fallers (*n* = 22)	Significance
Romberg test positive (*n*, %)	**6 (67%)**	**4 (18%)**	**0.015** ^a^
Reflexes absent or weak (*n*, %)	**7 (78%)**	**6 (27%)**	**0.017** ^a^
Vibration score (median, IQR)	**66, [45–94.5]**	**114, [87–132]**	**0.003** ^b^
Position sensing (median, IQR)	**5, [3–7.5]**	**9, [6.8–10]**	**0.002** ^b^
Two‐point discrimination (median, IQR)	**50, [40–50]**	**19, [10–30]**	**0.002** ^b^
MRC sum score (median, IQR)	**70, [67–72]**	**74, [73–78]**	**0.001** ^b^
Timed “Up & Go” test (median, IQR)	12, [9.8–16]	10.6, [8.6–12.9]	0.21^b^
DemTect (median, IQR)	14, [11–17]	15.5, [13–17.3]	0.40^b^

*Note*: *p*‐Values < .05 were estimated as significant and are highlighted in bold.

Abbreviations: IQR, interquartile range; MRC, Medical Research Council.

^a^ Chi‐square test.

^b^ *t*‐Test/Mann‐Whitney test.

### 3.4 Peripheral neuropathy as true risk factor for falls

**FIGURE 2:**
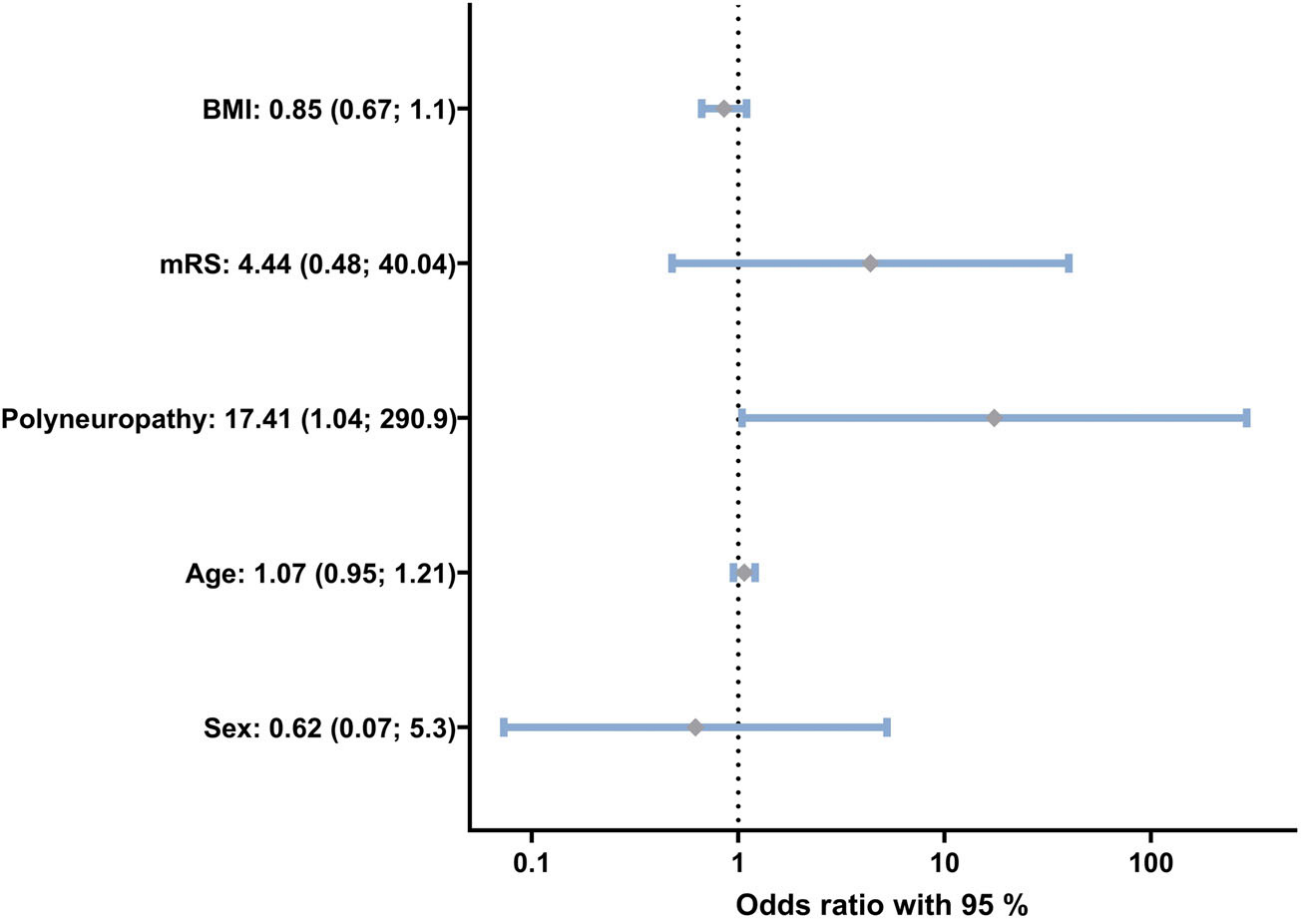
Binominal logistic regression analysis for the dependent variable falls. Odds ratio with 95% confidence interval (CI), given for each parameter on a log10‐scale. *p*‐Values for body mass index (BMI): .21, modified Rankin Scale (mRS): 0.19, polyneuropathy: 0.047, age: 0.28, sex: 0.66. Linear regression model: *R* ^2^: 0.459. Coding: BMI: Total score; mRS: 0—score of 0–1, 1—Score of 2–3; polyneuropathy: 0—no, 1—yes; age in years; sex: 0—female, 1—male.

In the next step, we aimed at identifying genuine demographic and clinical risk factors for falls. We excluded the variables of Tables 2 and 3 for this analysis, as the variables highly correlated with the variable polyneuropathy. We used a binomial multiple regression variable analysis (Figure 2). Only peripheral neuropathy was associated significantly with falls (odds ratio [OR] 17.41, *p*‐value: .047). Details of the model are given in Supplemental material 2.

**TABLE 3:** Subjective fall assessment and self‐questionnaires.

	Fallers (*n* = 9)	Non‐fallers (*n* = 22)	Significance
Feeling of increased stumbles (*n*, %)	**6 (67%)**	**3 (14%)**	**0.007** ^a^
Feeling of walking insecure (*n*, %)	**8 (89%)**	**8 (36%)**	**0.015** ^a^
Feeling of false estimation of heights (*n*, %)	4 (44%)	6 (27%)	0.41^a^
LUCAS Frailty Index (*n* of frail or pre‐frail, %)	4 (44%)	9 (41%)	1.00^a^
FES‐I (median, IQR)	**24, [19.5–27]**	**17.5, [16–22]**	**0.016** ^b^
DHI (median, IQR)	10, [4–31]	1, [0–32]	0.23^b^

*Note*: *p*‐Values < .05 were estimated as significant and are highlighted in bold.

Abbreviations: DHI, Dizziness Handicap Index; FES‐I, Falls Efficacy Scale Index; IQR, interquartile range.

^a^ Chi‐square test.

^b^ *t*‐Test/Mann‐Whitney test.

### 3.5 Fall assessment

Fallers reported significantly more often a feeling of increased stumbles (67%–14%, *p*‐value: .007) and insecure walking (89%–36%, *p*‐value: .015), while subjective height estimation remained comparable (*p*‐value: .41). The prevalence of frail and pre‐frail persons was comparable between fallers (44%) and non‐fallers (41%). Notably, the DHI questionnaire showed no differences between fallers and non‐fallers, indicating no subjective impact on daily activities; however, as indicated by the FES‐I questionnaire, fallers are concerned about falling (again) (see Table 3).

### 3.6 Computerized dynamic posturography

Technical assessment via computerized dynamic posturography was helpful in differentiating fallers and non‐fallers. The SOT showed no differences between the groups, but the Rhythmic Weight Shift test indicated a slower anterior to posterior on‐axis velocity (median in grad/s 3 to 3.5, *p*‐value: .023) and a lower bidirectional control in the faller group (median in % 60–76, *p*‐value: .015), indicating a higher forward/backward sway in these patients during voluntary movements (see Table 4). No differences were seen for the lateral movement. Stepping during mCTSIB was more frequently observed more for fallers (median 3) than non‐fallers (median 0, *p*‐value: .035). However, neither the composite score based on the center of gravity sway trajectory nor the percentual Limit of Stability in the mCTSIB was different between fallers and non‐fallers. When comparing the polyneuropathy patients with controls, both the mCTSIB and the Rhythmic Weight Shift test could differentiate polyneuropathy patients from controls. The mCTSIB composite score distinguished older neuropathy patients (median 1.5) from older controls (median 0.8, *p*‐value: .022). Both lateral and anterior‐posterior voluntary movements were impaired (Supplemental material 1d).

**TABLE 4:** Dynamic computerized posturography.

	Fallers (*n* = 8)	Non‐fallers (*n* = 20)	Significance
mCTSIB (median, IQR)			
Overall	1.5, [0.9–2.9]	0.8, [0.7–1.9]	0.06^a^
Eo firm	0.3, [0.2–0.6]	0.3, [0.2–0.4]	0.35^a^
Ec firm	0.4, [0.2–0.8]	0.4, [0.2–0.6]	0.8^a^
Eo foam	0.9, [0.8–1.2]	0.9, [0.7–1.3]	0.76^a^
Ec foam	4.4, [1.8–6]	2.0, [1.4–5.5]	0.21^a^
Limits of stability (mean, SD)	56.4, ±17.4	58.8, **±**15.1	0.37^a^
Steps (median, IQR)	**3, [0–5.3]**	**0, [0–2.3]**	**0.035** ^a^
SOT (median, IQR)			
SOT composite Score	85, [73.5–90.3]	75.5, [66.8–82.8]	0.13^a^
Steps	0.5, [0–2.8]	0, [0–1]	0.126^a^
Motor control tests			
Rhythmic Weight Shift test (median, IQR)			
Anterior‐posterior on‐axis velocity (deg/s)	**3, [2.5–3.1]**	**3.5, [2.9–3.8]**	**0.023** ^a^
Anterior bidirectional control (%)	**60.1, [51–74]**	**76, [67–79.8]**	**0.015** ^a^
Lateral on‐axis velocity (degree/s)	4.8, [3.7–4.8]	5, [3.8–6.1]	0.26^a^
Lateral bidirectional control (%)	78, [70–82]	77.5, [74.3–80.8]	0.74^a^
Adaptation test (median, IQR)			
Slips	0, [0–4]	0, [0–1.8]	0.69^a^

*Note*: *p*‐Values < .05 were estimated as significant and are highlighted in bold.

Abbreviations: Ec firm, eyes closed and firm surface; Ec foam, eyes closed and unstable surface; Eo firm, eyes open and firm surface; Eo foam, eyes open and unstable surface; IQR, interquartile range; mCTSIB, modified Clinical Test of Sensory Interaction on Balance; SD, standard deviation; SOT, Sensory Organization Test.

^a^ *t*‐Test/Mann‐Whitney test.

### 3.7 Multiple linear regression analysis

**FIGURE 3:**
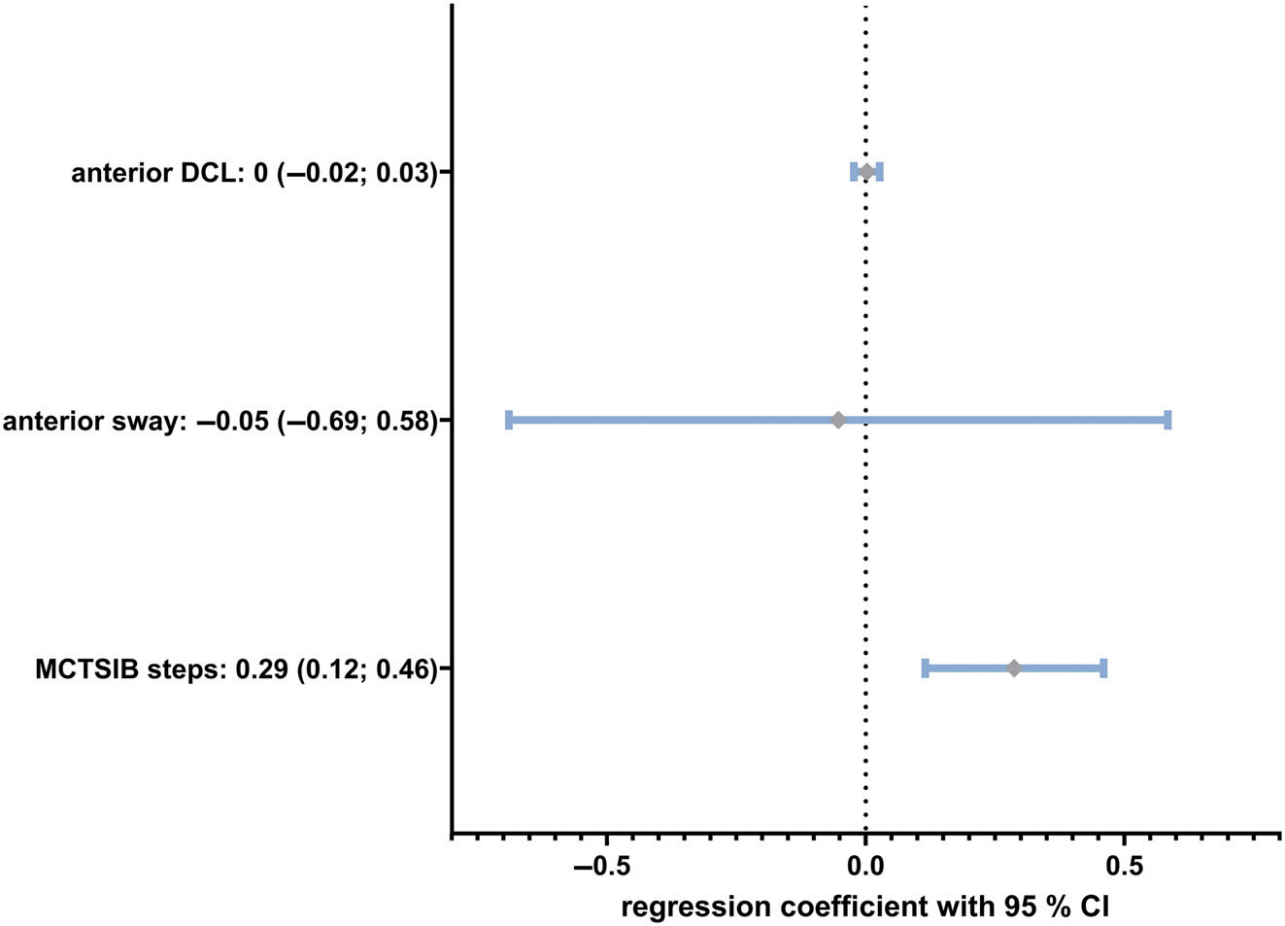
Multivariate linear regression analysis for the dependent variable number of falls in the last year. Odds ratio with 95% confidence interval (CI) for the regression coefficient, given for each parameter on a linear scale. *p*‐Values for anterior to posterior bidirectional control in % (DCL): 0.88, anterior to posterior on‐axis velocity: 0.87, modified Clinical Test of Sensory Interaction in Balance (MCTSIB) steps: 0.002.

In the next step, we wanted to evaluate which of the computerized dynamic posturography‐derived variables could predict high‐risk fallers. We used the overall number of falls in the last year as a dependent variable in a linear multiple regression analysis and the variables reaching statistical significance as independent variables. Our model showed a robust prediction of *R* ^2^ = 0.433, *p*‐value: .003 (details of the model are given in Supplemental material 3). The only variable significantly adding to the prediction was the recorded number of steps in the mCTSIB (standardized beta‐coefficient of 0.653, *p*‐value: .002). The unstandardized beta‐coefficients with its confidence intervals are plotted in Figure 3.

## 4 DISCUSSION

Our data align with and extend previous work suggesting that peripheral neuropathy is a genuine risk factor for falls. We applied rigorous exclusion and inclusion criteria: All older subjects included were home‐dwelling seniors with comparable demographic characteristics. Nearly all falls occurred in the neuropathy group (88%). Richardson and Hurvitz demonstrated that sensorimotor neuropathy of the lower limbs was associated with increased falls.^17^ However, in that study stratification for possible confounds was lacking. A complex and multilayered sensorimotor network enables postural stability, and controlling for potential confounds (e.g., impaired vestibular input) is crucial to understanding the reasons for falls. Older persons often display multiple potential risk factors, making the assessment even more complex. We, therefore, extensively characterized our patients for other confounds and reported risk factors for falls (e.g., timed “Up & Go” test, comorbidities, cognitive function, and exclusion of vestibulocochlear affection). In controls and patients with peripheral neuropathy, nerve conduction studies were performed to exclude or confirm polyneuropathy of the lower limbs. Multiple variable binomial regression analysis showed that only peripheral neuropathy was significantly associated with falls compared with other epidemiological parameters and increased the odds in our elderly home‐dwelling cohort over 16 times fold. Richardson and Hurvitz reported a similar OR of 17.^17^ By stark contrast, two other large epidemiological studies reported ORs between 1.5 and 2.5.^18^, ^48^

Computerized dynamic posturography allows quantification of balance and its disturbances in healthy subjects and pathological conditions, for example, Parkinson's disease.^42^, ^45^ It detected elderly fallers,^49^ and the SOT detected subclinical affection in patients with electrophysiological diabetic peripheral neuropathy. Body sway has recently been described as an objective parameter to detect treatment response in chronic inflammatory demyelinating polyneuropathy (CIDP).^50^, ^51^ In our study, only the mCTSIB and Rhythmic Weight Shift test, not the SOT and the adaption test, differentiated polyneuropathy patients from controls. Several variables distinguished polyneuropathy patients from controls; however, fallers were only identified by impairment of voluntary control for anterior‐posterior bidirectional movements and the number of steps during mCTSIB. The number of steps correlated as the only variable significantly with the overall number of falls, indicating that the number of steps might be of particular interest in quantifying the fall risk in older persons, particularly in older neuropathy patients, enabling personalized preventive therapies. A step forward in conditions challenging postural control is termed the “stepping strategy.”^52^ This strategy brings the center of mass closer to the base of the support, correlating with the feet in upright humans, stabilizing balance and preventing a fall.^53^, ^54^ Our results indicate that this coping mechanism is readily applied in challenging postural situations in polyneuropathy patients and that its use correlates with the overall fall risk. Stepping in natural situations occurs mainly in an anterior‐posterior direction, but lateral stepping is another coping mechanism to maintain postural control.^55^ Within a specific range, a wider gait and stance by moving the feet more apart can prevent falls.^56^ In both our polyneuropathy patients and in fallers, only anterior‐posterior voluntary postural control was impaired. Taken together, our results suggest that stepping is a coping mechanism in older polyneuropathy patients and that impaired control over this mechanism is associated with a higher number of falls. This aligns with reported decreased accuracy of voluntary stepping on a walkway of diabetic neuropathy patients.^57^

Reasons for this loss of voluntary movement control can be derived from our cohort's characteristics: the significantly lower MRC sum score reflects diminished muscle control that is critical in the swing phase, when the center of mass is balanced on one leg, and the initial stance phase, when the center of mass is shifted.^53^ Further, peripheral polyneuropathy affected proprioception, as indicated by a lower vibration score and absent tendon reflexes, resulting in impaired muscle fine‐tuning and agonist‐antagonist interplay. Decreased touch acuity was also present in the fallers, adding to the sensory impairment.

Our study has three critical limitations that should be addressed in future studies. First, we only assessed fall incidence retrospectively and, thus, relied on self‐reports. Underreporting of falls has been reported in geriatric patients.^58^, ^59^ Second, we included multiple neuropathy entities and neurophysiological subtypes in this study. Especially in immune‐mediated neuropathies, fluctuating symptoms have been observed.^51^ We accounted for this potential confound by including only patients who were clinically stable over the last year and had no treatment change. Third, computerized dynamic posturography is a technical assessment that may not be available in most clinical settings, limiting its potential as a screening tool. Our study indicates that a sensitive bedside test that assesses both loss of somatosensory input and the voluntary adapting motor control would be needed to identify elderly at risk for falls.

In summary, our study highlights the urgent need for fall prevention in older polyneuropathy patients and the need for a structured assessment of the individual fall risk. Our data suggest that computerized dynamic posturography is helpful for quantifying fall risk. Further, our results implicate that a potential target for preventing and rehabilitating polyneuropathy patients with postural instability is the stepping strategy and its control via the voluntary anterior‐posterior movement during upright gait and stance.

## AUTHOR CONTRIBUTIONS

FK and HCL designed and conceptualized the study. FK and CS and interpreted the data. FK, CS, H‐DK, DW, MS, and HCL gathered the data. FK drafted the manuscript. All authors critically reassessed the study and revised the final version of the manuscript.

## FUNDING INFORMATION

FK was supported by the Cologne Clinician Scientist Program (CCSP)/Faculty of Medicine/University of Cologne. Funded by the Deutsche Forschungsgemeinschaft (DFG, German Research Foundation) (Project No. 413543196).

## CONFLICT OF INTEREST STATEMENT

FK received travel reimbursement from Alexion, not related to this work. GW has received advisory board honoraria and speaker fees from Alnylam, Biogen, Hormosan, Pfizer, and Sobi outside of the submitted work. MS has received speaker honoraria from Alexion Pharmaceuticals, argenx, Bayer, Biogen, CSL Behring, Genzyme, Grifols, Merck, Miltenyi Biotec, Novartis, Roche, Teva, and Hormosan Pharma. He is vice chairman of the medical advisory board of the German Myasthenia Gravis Society. GRF received royalties from Springer, Thieme, and Hogrefe. He declared speaker honoraria from Forum für medizinische Fortbildung (FomF) GmbH and the Deutsche Gesellschaft für Neurologie (DGN). HCL received honoraria for speaking and advisory board engagement or academic research support by Akcea, Alnylam, Biogen, Celgene, CSL Behring, Grifols, Gruenenthal, LFB Pharma, Takeda, and UCB. The remaining authors report no conflicts of interest.

## Supporting information


**Data S1.** Supporting information.

## Data Availability

The data that support the findings of this study are available on request from the corresponding author. The data are not publicly available due to privacy or ethical restrictions.
