# Supplementary material for: Peripheral neuropathy, an independent risk factor for falls in the elderly, impairs stepping as a postural control mechanism: A case‐cohort study
Source: J Peripher Nerv Syst. 2024 Sep 1;29(4):453–63. doi: 10.1111/jns.12656 (PMC11625983; doi:10.1111/jns.12656)
Supplement: Supplementary file 1 — Data S1. Supporting information. [file JNS-29-453-s001.docx]

**Supplemental material (online-only)**

**Supplemental material 1***. a) Patients’ characteristics of polyneuropathy patients and elderly controls.*

|  | Polyneuropathy  (n=17) | No polyneuropathy (n=14) | Significance |
| --- | --- | --- | --- |
| Sex, (n of females) | 5 (29 %) | 9 (64 %) | 0.16† |
| Age (mean in years, SD) | 70.7, ± 8.3 | 71.6, ± 8.9 | 0.78‡ |
| BMI (mean, SD) | 28.4, ± 5.6 | 25.2, ± 5.1 | 0.1‡ |
| Polyneuropathy entity (n) | Inflammatory: 6 (35 %)  *Definite CIDP (n=3)*  *NSVN (n=3)*  Diabetic: 4 (24 %)  Idiopathic: 7 (41 %) |  |  |
| **mRS, (median, IQR)** | **1, [0 – 2]** | **0, [0 – 1]** | **0.036‡** |
| Comorbidities, (n)  Congestive heart Failure  Hemiplegia or paraplegia  Dementia  Chronic pulmonary disease  Rheumatological disease  Diabetes with chronic complications  Renal disease  Any malignancy  Metastatic solid tumor  Liver disease, any  Acquired immunodeficiency syndrome  Charlson comorbidity index (max. 24, median, IQR) | 2 (12 %)  0 (0 %)  0 (0 %)  2 (12 %)  2 (12 %)  4 (24 %)  1 (6 %)  1 (6 %)  1 (6 %)  0 (0 %)  0 (0 %)  0, [0 – 1.5] | 1 (7 %)  0 (0 %)  0 (0 %)  2 (14 %)  2 (14 %)  1 (7 %)  1 (7 %)  0 (0 %)  0 (0 %)  0 (0 %)  0 (0 %)  1, [0 – 2] | 0.14‡ |
| Medication, (n)  Antihypertensives  Antipsychotics  Antidepressants  Anxiolytics  Narcotics  Nonsteroidal anti-inflammatory drugs  Antihistamines  Anticholinergic drugs  Antiepileptics  Proton pump inhibitors | 6 (35 %)  0 (0 %)  3 (18 %)  0 (0 %)  2 (12 %)  1 (6 %)  0 (0 %)  1 (6 %)  2 (12 %)  4 | 9 (64 %)  0 (0 %)  1 (7 %)  0 (0 %)  0 (0 %)  0 (0 %)  0 (0 %)  0 (0 %)  1 (7 %)  1 |  |
| N. suralis  **sNCV (median in m/s, IQR)**  **SNAP (median in µV, IQR)** | **34, [0 – 39]**  **0.9, [0 – 4]** | **42.8, [40 – 46]**  **12.5, [6.8 – 17.3]** | **<0.001‡**  **<0.001‡** |
| N. tibialis  **mNCV (median in m/s, IQR)**  **CMAP (median in mV, IQR)**  **DML (median in m/s, IQR)** | **39, [34.5 – 43.5]**  **3, [0.7 – 7.1]**  **4.9, [4.3 – 5.9]** | **46, [42 – 53.5]**  **13, [9.5 – 17.6]**  **3.8, [3.4 – 4.1]** | **<0.001‡**  **<0.001‡**  **<0.001‡** |
| Polyneuropathy subtype by NCS (n)  Mainly axonal sensorimotor  Mainly axonal sensory  Axonal-demyelinating sensorimotor | 4 (24 %)  4 (24 %)  9 (53 %) |  |  |

Abbreviations: BMI: body mass index, mRS: modified Rankin Scale. sNCV: sensory nerve conduction velocity, SNAP: sensory nerve action potential, mNCV: motor nerve conduction velocity, CMAP: compound muscle action potential. DML: distal motor latency, SD: standard deviation, IQR: interquartile range, NCS: nerve conduction studies, †: chi-square test, **‡:** t-Test / Mann-Whitney test.

*b) Neurological examination, including gait and sensorimotor assessment of polyneuropathy patients versus controls.*

|  | Polyneuropathy  (n=17) | No polyneuropathy (n=14) | Significance |
| --- | --- | --- | --- |
| **Romberg positive (n, %)** | **9 (53 %)** | **1 (7 %)** | **0.009†** |
| **Reflexes absent or weak (n, %)** | **11 (65 %)** | **2 (14 %)** | **0.009†** |
| **Vibration score (median, IQR)** | **66, [48 – 93]** | **124.5, [107 – 133]** | **<0.001‡** |
| Position sensing (median, IQR,) | 8, [4 – 9] | 9, [5 – 10] | 0.09‡ |
| **Two-point discrimination**  **(median in mm, IQR)** | **50, [17 – 50]** | **20, [10 – 26.3]** | **0.026‡** |
| **MRC sum score (median, IQR)** | **73, [69 – 75.5]** | **74, [73.8 – 78.5]** | **0.029‡** |
| Timed “Up & Go” test (median, IQR) | 12, [9.4 – 15.5] | 10.3, [8,9 – 12] | 0.20‡ |
| DemTect (median, IQR) | 15, [11 – 17] | 15, [13 – 18] | 0.58‡ |

Abbreviations: SD: standard deviation, IQR: interquartile range, †: chi-square test, **‡:** t-Test / Mann-Whitney test.

*c): Subjective fall assessment and self-questionnaires.*

|  | Polyneuropathy  (n=17) | No polyneuropathy (n=14) | Significance |
| --- | --- | --- | --- |
| **Fallers (n, %)** | **8 (47 %)** | **1 (7 %)** | **0.021†** |
| **Number of falls in the last year (median, IQR)** | **0, [0 – 1.5]** | **0, [0 – 0]** | **0.014‡** |
| **Feeling of increased stumbles (n, %)** | **8 (47 %)** | **1 (7 %)** | **0.021†** |
| **Feeling of walking insecure (n, %)** | **12 (71 %)** | **4 (29 %)** | **0.032†** |
| Feeling of false estimation of heights (n, %) | 8 (47 %) | 2 (14 %) | 0.07† |
| LUCAS-Frailty- Index (n of frail or pre-frail, %) | 8 (47 %) | 5 (36 %) | 0.72† |
| **FES-I (median, IQR)** | **23, [18 – 27]** | **16.5, [16 – 20]** | **0.003‡** |
| DHI (median, IQR) | 10, [0 – 36] | 1, [0 – 11] | 0.17‡ |

Abbreviations: FES-I: Falls Efficacy Scale Index, DHI: Dizziness Handicap Index, SD: standard deviation, IQR: interquartile range, †: chi-square test, **‡:** t-Test / Mann-Whitney test.

*d): Dynamic computerized posturography of polyneuropathy patients versus controls.*

|  | Polyneuropathy  (n=16) | No polyneuropathy (n=12) | Significance |
| --- | --- | --- | --- |
| mCTSIB (median, IQR)  **overall**  Eo firm  Ec firm  Eo foam  **Ec foam**  Limits of Stability (mean, SD)  Steps (median, IQR) | **1.5, [0.8 – 2.1]**  0.3, [0.2 – 0.6]  0.5, [0,2 – 0.9]  0.9, [0.8 – 1.3]  **2.7, [1.8 – 6]**  54.9, **±** 15.3  1.5, [0 – 3] | **0.8, [0.7 - 1.3]**  0.3, [0.2 – 0.6]  0.3, [0.2 – 0.6]  0.9, [0.7 – 1.2]  **1.8, [1.3 – 3.5]**  62.4, **±** 15.4  0, [0 – 0] | **0.022**‡  0.55‡  0.24‡  0.94‡  **0.037**‡  0.21‡  0.06**‡** |
| SOT (median, IQR)  SOT Composite Score  SOM  VIS  VEST  PREF  Steps | 79, [67 – 88]  95.5, [92.3 – 98.8]  83, [82 – 87.8]  68, [62.3 – 73.8]  97, [86,8 – 100]  0, [0 – 1] | 77, [70 – 82]  94, [83.5 – 98]  88, [76.5 – 94.3]  73.5, [58.3 – 82.3]  100, [95.3 – 100]  0, [0 – 1] | 0.47‡  0.2‡  0.5‡  0.36‡  0.4‡  0.6‡ |
| Motor control tests  Rhythmic Weight Shift test (median, IQR)  **Anterior-posterior on-axis velocity (deg/sec)**  **Anterior bidirectional control (%)**  **Lateral on-axis velocity (deg/sec)**  Lateral bidirectional control (%)  Adaptation test (median, IQR)  Slips | **3, [2.6 – 3.2]**  **65.5, [52.8 – 74.5]**  **4.5, [3.5 – 5]**  **76, [70 – 81.5]**  1.6, **±** 2.2 | **3.3, [3.6 – 4.1]**  **79, [74.5 – 81.5]**  **5.3, [4.6 – 6.7]**  **80, [76.3 – 82.5]**  0.8 **±** 2.4 | **0.003**‡  **0.003‡**  **0.046‡**  0.08  0.15‡ |

Abbreviations: mCTSIB: modified Clinical Test of Sensory Interaction on Balance, Eo firm: eyes open and firm surface, Ec firm: eyes closed and firm surface, Ec foam: eyes open and unstable surface, Ec foam: eyes closed and unstable surface, SOT: Sensory Organization Test, SOM: sensory analysis ratio for somatosensory input, VIS: sensory analysis ratio for visual input, VEST: sensory analysis ratio for vestibular input, PREF: sensory analysis ratio for (incorrect) visual input, SD: standard deviation, IQR: interquartile range, **‡:** t-Test / Mann-Whitney test.

**Supplemental material 2: Details of the binominal regression model.**

Regression coefficient B: -.894

Standard error: .396

Df: 5.103

Significance: 0.024

Exp(B): 4.09

|  | | Regression-coefficient B | Standard error | Wald | df | Sig. | Exp(B) | 95% confidence-intervall EXP(B) | |
| --- | --- | --- | --- | --- | --- | --- | --- | --- | --- |
|  |  |  |  |  |  |  |  | Lower value | Upper value |
|  | Gender(1) | -.476 | 1.090 | .191 | 1 | .662 | .621 | .073 | 5.260 |
|  | Age | .066 | .062 | 1.157 | 1 | .282 | 1.069 | .947 | 1.206 |
|  | Polyneuropathy(1) | 2.857 | 1.437 | 3.954 | 1 | .047 | 17.407 | 1.042 | 290.902 |
|  | mRS(1) | 1.475 | 1.130 | 1.706 | 1 | .192 | 4.373 | .478 | 40.035 |
|  | BMI | -.158 | .128 | 1.542 | 1 | .214 | .854 | .665 | 1.096 |
|  | constant | -3.563 | 5.564 | .410 | 1 | .522 | .028 |  |  |

| *Correlation matrix* | | | | | | | |
| --- | --- | --- | --- | --- | --- | --- | --- |
|  | | constant | Gender(1) | Age | Polyneuropathy(1) | mRS(1) | BMI |
|  | constant | 1.000 | -.094 | -.820 | -.222 | -.045 | -.458 |
|  | Gender(1) | -.094 | 1.000 | .036 | .319 | -.256 | -.112 |
|  | Age | -.820 | .036 | 1.000 | .275 | .103 | -.075 |
|  | Polyneuropathy(1) | -.222 | .319 | .275 | 1.000 | -.048 | -.372 |
|  | mRS(1) | -.045 | -.256 | .103 | -.048 | 1.000 | -.111 |
|  | BMI | -.458 | -.112 | -.075 | -.372 | -.111 | 1.000 |

Abbreviations: mRS: modified Rankin Scale, BMI: body mass index.

**Supplemental material 3: Details of the multivariate linear regression model.**

| *ANOVA* | | | | | | |
| --- | --- | --- | --- | --- | --- | --- |
| Model | | Square sum | df | Mean of squares | F | Sig. |
|  | regression | 8.203 | 3 | 2.734 | 6.098 | .003^b^ |
|  | unstandardized  residual | 10.761 | 24 | .448 |  |  |
|  | total | 18.964 | 27 |  |  |  |
| a. dependent variable: falls | | | | | | |
| b. included variables: MCTSIB_steps, anteriordcl, anteriorsway | | | | | | |

| Model | | Unstandardized  coefficient | | Standardized coefficient | T | Sig. |
| --- | --- | --- | --- | --- | --- | --- |
|  |  | Regression coefficient B | Standard error | Beta |  |  |
|  | constant | .136 | .870 |  | .156 | .877 |
|  | anteriorsway | -.053 | .309 | -.042 | -.172 | .865 |
|  | anteriordcl | .002 | .012 | .037 | .159 | .875 |
|  | MCTSIB_steps | .287 | .084 | .653 | 3.439 | .002 |

|  | 95% CI for B |  |  |  |
| --- | --- | --- | --- | --- |
|  | Lower value | Upper value | Tolerance | VIF |
| constant | -1.661 | 1.932 |  |  |
| anteriorsway | -.690 | .584 | .405 | 2.468 |
| anteriordcl | -.023 | .027 | .439 | 2.277 |
| MCTSIB_steps | .115 | .460 | .657 | 1.523 |

Abbreviations: anteriordcl: Anterior bidirectional control, anteriorsway: Anterior-posterior on-axis velocity (deg/sec), MCTSIB: modified Clinical Test of Sensory Interaction on Balance, VIF: variance inflation factor, CI: confidence interval.
